# Look Before You Cut: How a Killian–Jamieson Diverticulum Can Lead to Unnecessary Thyroidectomy

**DOI:** 10.1002/kjm2.70141

**Published:** 2025-11-20

**Authors:** Yi‐Wei Sun, Tzu‐Yen Huang, Che‐Wei Wu

**Affiliations:** ^1^ Department of Otorhinolaryngology, Kaohsiung Medical University Hospital Kaohsiung Medical University Kaohsiung Taiwan; ^2^ Department of Otorhinolaryngology, School of Post‐Baccalaureate Medicine, College of Medicine Kaohsiung Medical University Kaohsiung Taiwan; ^3^ Department of Otolaryngology—Head and Neck Surgery Kaohsiung Medical University Gangshan Hospital, Kaohsiung Medical University Kaohsiung Taiwan; ^4^ Department of Otorhinolaryngology, School of Medicine, College of Medicine Kaohsiung Medical University Kaohsiung Taiwan

The increasing use of ultrasonography in thyroid screening has significantly improved the detection of thyroid malignancies, but it also poses new diagnostic challenges, particularly the incidental discovery of neck masses that appear to mimic thyroid nodules. A rare but clinically important pitfall is the Killian–Jamieson diverticulum (KJD), a pharyngoesophageal outpouching that can closely resemble a suspicious thyroid nodule. Herein, we present a case that underscores the importance of clinical vigilance to prevent misdiagnosis and avert unwarranted interventions. KJD originates in the anterolateral cervical esophagus, in contrast to the more common posterior Zenker's diverticulum [1]. Its proximity to the thyroid gland, particularly the left lobe, allows KJD to present as a palpable mass and trigger a thyroid workup. On ultrasonography, KJD often appears as a hypoechoic lesion with punctate echogenic foci, mimicking the microcalcifications typically associated with papillary thyroid carcinoma [2]. Consequently, KJD may meet the criteria for fine‐needle aspiration biopsy (FNAB). Unintentional aspiration of a diverticulum can yield squamous cells, debris, or even food particles, thereby complicating cytologic interpretation and potentially leading to misclassification within the Bethesda System [3].

We present the case of a 46‐year‐old woman illustrating this diagnostic dilemma. She was referred by an endocrinologist for surgical management of a suspicious thyroid nodule classified as TI‐RADS 4, with FNAB showing atypia of undetermined significance (Figure 1, panel A, arrow). A computed tomography (CT) scan revealed intralesional air within a paracervical esophageal lesion (panel B), raising suspicion of a diverticulum. We then proceeded with barium esophagography (panel C), which confirmed a left‐sided diverticular outpouching rather than a thyroid lesion, thereby sparing the patient from unnecessary invasive procedures. This case illustrates the limitations of relying solely on ultrasound and cytology for thyroid lesions and highlights the essential role of adjunct imaging in securing the correct diagnosis.

**FIGURE 1:**
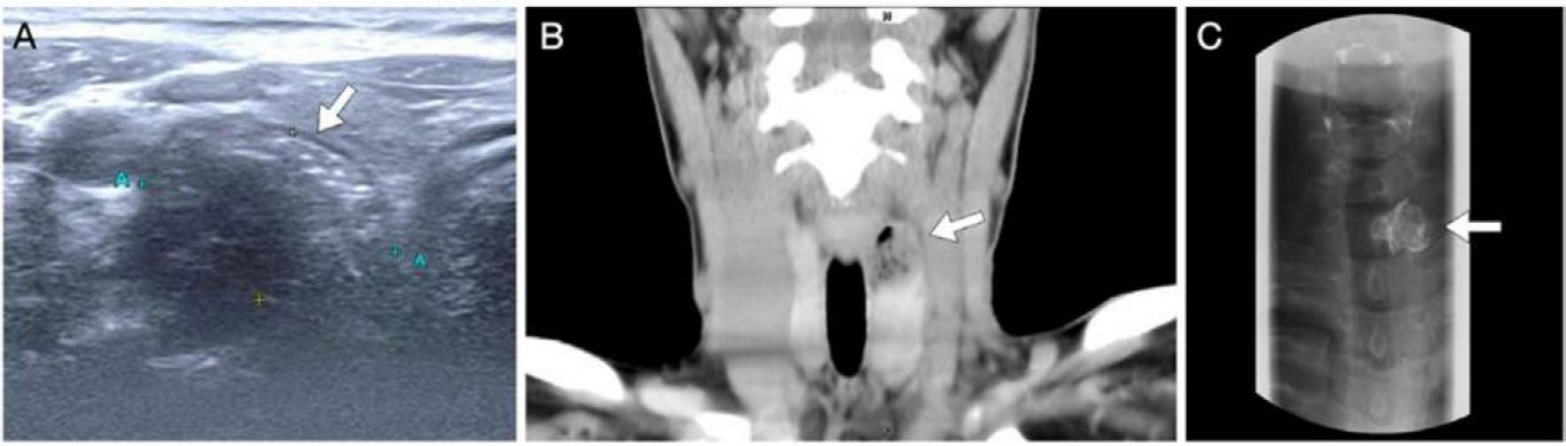
Imaging findings of Killian–Jamieson diverticulum mimicking a thyroid nodule. (A) Ultrasonography showing a 2.1‐cm hypoechoic lesion with punctate echogenic foci (arrow) in the upper pole of the left thyroid lobe, initially suspicious for malignancy. (B) Coronal computed tomography scan demonstrating intralesional air within a paracervical esophageal lesion (arrow). (C) Barium esophagography confirming a left anterolateral cervical esophageal outpouching (arrow), consistent with Killian–Jamieson diverticulum.

The consequences of misdiagnosing KJD are significant. Previous studies have shown that KJD is frequently mistaken for a thyroid lesion on ultrasonography. In the largest review to date, Haddad et al. reported that 35% of cases were initially misdiagnosed as thyroid nodules [4]. An unnecessary thyroidectomy exposes patients to surgical risks and places the recurrent laryngeal nerve (RLN) in jeopardy due to the diverticulum's location within the tracheoesophageal groove. Asymptomatic KJD can be safely managed with observation, making accurate recognition paramount.

A systematic diagnostic strategy is therefore crucial. Clinicians should consider KJD in the differential diagnosis whenever ultrasonography reveals a suspicious nodule, particularly near the upper pole of either thyroid lobe. KJD typically appears as a heterogeneous hypoechoic lesion posterior to the thyroid lobe with internal echogenic foci caused by intraluminal air rather than true calcifications. Unlike a true thyroid nodule, which maintains a fixed echotexture, KJD demonstrates dynamic changes in contour during swallowing, suggesting an esophageal origin, while Doppler exhibits no vascularity [5]. These features are valuable for distinguishing KJD from thyroid nodules. Dynamic ultrasonography offers a first clue, and if uncertainty persists, cross‐sectional imaging or barium esophagography must precede any invasive procedure to confirm the diagnosis and guide appropriate management.

In conclusion, KJD is an uncommon but clinically relevant mimic of thyroid nodules. By maintaining clinical vigilance and utilizing dynamic and CT imaging, clinicians can prevent misdiagnosis, avoid unnecessary surgery, and minimize serious complications that could follow.

## Conflicts of Interest

The authors declare no conflicts of interest.

## Data Availability

Data sharing not applicable to this article as no datasets were generated or analysed during the current study.
